# Supplementary material for: Causal Relationship between Aging and Anorexia Nervosa: A White-Matter-Microstructure-Mediated Mendelian Randomization Analysis
Source: Biomedicines. 2024 Aug 16;12(8):1874. doi: 10.3390/biomedicines12081874 (PMC11351342; doi:10.3390/biomedicines12081874)
Supplement: Supplementary file 1 [file biomedicines-12-01874-s001.zip › Supplementary Figure/Figure S4/Singe SNP plot of MR analysis for TL to ubm-b-1995.pdf]

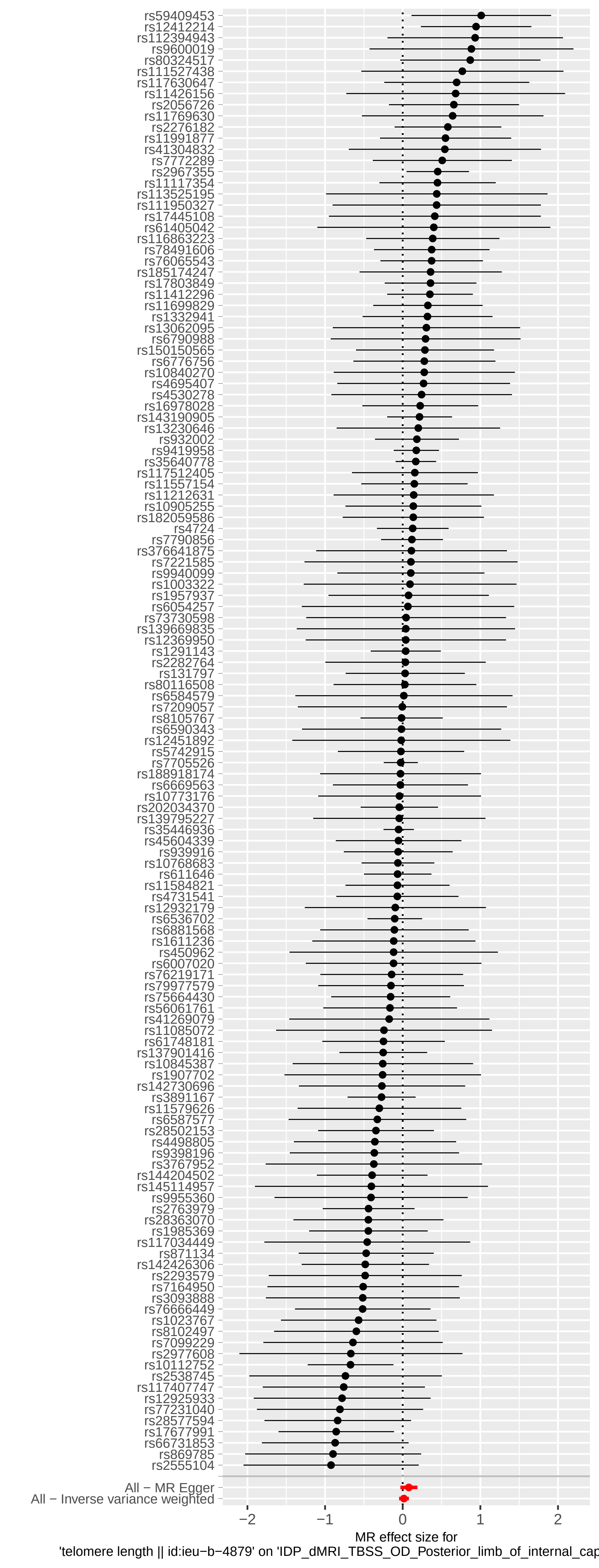

rs59409453
rs12412214
rs112394943
rs9600019
rs80324517
rs111527438
rs117630647
rs11426156
rs2056726
rs11769630
rs2276182
rs11991877
rs41304832
rs7772289
rs2967355
rs11117354
rs113525195
rs111950327
rs17445108
rs61405042
rs116863223
rs78491606
rs76065543
rs185174247
rs17803849
rs11412296
rs11699829
rs1332941
rs13062095
rs6790988
rs150150565
rs6776756
rs10840270
rs4695407
rs4530278
rs16978028
rs143190905
rs13230646
rs932002
rs9419958
rs35640778
rs117512405
rs11557154
rs11212631
rs10905255
rs182059586
rs4724
rs7790856
rs376641875
rs7221585
rs9940099
rs1003322
rs1957937
rs6054257
rs73730598
rs139669835
rs12369950
rs1291143
rs2282764
rs131797
rs80116508
rs6584579
rs7209057
rs8105767
rs6590343
rs12451892
rs5742915
rs7705526
rs188918174
rs6669563
rs10773176
rs202034370
rs139795227
rs35446936
rs45604339
rs939916
rs10768683
rs611646
rs11584821
rs4731541
rs12932179
rs6536702
rs6881568
rs1611236
rs450962
rs6007020
rs76219171
rs79977579
rs75664430
rs56061761
rs41269079
rs11085072
rs61748181
rs137901416
rs10845387
rs1907702
rs142730696
rs3891167
rs11579626
rs6587577
rs28502153
rs4498805
rs9398196
rs3767952
rs144204502
rs145114957
rs9955360
rs2763979
rs28363070
rs1985369
rs117034449
rs871134
rs142426306
rs2293579
rs7164950
rs3093888
rs76666449
rs1023767
rs8102497
rs7099229
rs2977608
rs10112752
rs2538745
rs117407747
rs12925933
rs77231040
rs28577594
rs17677991
rs66731853
rs869785
rs2555104
All − MR Egger
All − Inverse variance weighted
−2
−1
0
1
2
MR effect size for
'telomere length || id:ieu−b−4879' on 'IDP_dMRI_TBSS_OD_Posterior_limb_of_internal_cap
